# Preliminary Antibacterial Activity Against *Staphylococcus epidermidis* and Chemical Profile of *Gracilaria caudata* Crude Methanolic Extract

**DOI:** 10.1155/ijm/1166420

**Published:** 2026-10-22

**Authors:** Mariana de Sousa Santos Hempel, Michael dos Anjos dos Santos, Richard Coelho de Araujo dos Santos, João Vitor dos Anjos Reis, Leonardo Gripp Bom Amorim, Bruno Francesco Rodrigues de Oliveira, Leonardo Zambotti-Villela, Lohengrin Dias de Almeida Fernandes, Sávio Henrique Calazans Campos, Pio Colepicolo Neto, Ricardo Coutinho

**Affiliations:** ^1^ Department of Marine Biotechnology Sea Studies Institute Admiral Paulo Moreira Arraial do Cabo Rio de Janeiro Brazil; ^2^ Division of Chemical Oceanography and Environmental Geochemistry Sea Studies Institute Admiral Paulo Moreira Arraial do Cabo Rio de Janeiro Brazil; ^3^ Department of Microbiology and Parasitology Biomedical Institute Federal Fluminense University Niterói Rio de Janeiro Brazil uff.br; ^4^ Department of Biochemistry Chemistry Institute University of São Paulo São Paulo Brazil usp.br

**Keywords:** antibacterial, human health, macroalgae, methanolic extract, Rhodophyta, *Staphylococcus epidermidis*

## Abstract

Currently, the dilemma of antimicrobial resistance (AMR) worsens each year and poses a threat to global health. Macroalgae are potentially excellent sources of highly bioactive secondary metabolites, particularly useful for developing new functional ingredients, and might represent a promising reservoir of novel antimicrobial agents in the fight against AMR. This study was conducted to determine whether methanolic extracts from *Gracilaria caudata* (MeOHGc) macroalgae could serve as potential alternatives against human pathogenic bacteria. The antimicrobial activity of MeOHGc was evaluated using the agar disc diffusion method against gram‐positive *Staphylococcus epidermidis* and gram‐negative *Escherichia coli*. The chemical profile of the methanolic extract was assessed by qualitative phytochemical screening and gas chromatography–mass spectrometry (GC/MS) analysis. The results of phytochemical tests revealed the presence of sterols, terpenoids, tannins, and polyphenols. The GC‐MS profile of MeOHGc putatively identified two chemical constituents based on the pre‐established similarity index threshold (SI ≥ 90%): benzenepropanoic acid, 3,5‐bis (1,1‐dimethylethyl)‐4‐hydroxy‐methyl ester (SI: 96.8%) and phenol, 2,4‐bis (1‐methyl‐1‐phenylethyl)‐ (SI: 96.8%). The antimicrobial activity assay showed preliminary antibacterial activity against *S. epidermidis* at the tested concentrations, with no activity detected against *E. coli*. Our results place *G. caudata* as a potential source worth investigating for the development of new antimicrobial agents, and open perspectives for its further investigation as biomolecular sources of these compounds.

## 1. Introduction

The dilemma of antimicrobial resistance worsens each year and poses a threat to global health. Over the coming decades, expectations point to a significant increase in deaths from antimicrobial‐resistant microorganisms, even surpassing those from cancer, and the likely cumulative scenario anticipates approximately 40 million deaths by 2050 [1]. Represented among the priority pathogens for research and development of new antimicrobial agents [2], *Staphylococcus* species are among the main pathogens responsible for a multitude of health‐associated and community infections with varying degrees of severity that can threaten human life and cause death. For example, the coagulase‐negative bacterium *Staphylococcus epidermidis* is a serious opportunistic pathogen that causes skin diseases and implant‐associated infections, leading to nosocomial bloodstream infections, often with complications and poor prognosis [3]. Lately, infections caused by *S. epidermidis* have attracted attention due to an increase in cases involving strains resistant to methicillin and other antibiotic classes [4], with resistance to last‐resort antimicrobial drugs against multidrug‐resistant *S. epidermidis* increasing at a daunting pace both in clinical and veterinary settings [5, 6]. In parallel, the emergence of resistant strains represents a critical challenge for global public health, owing to limited therapeutic options and the associated increased morbidity and mortality [7].

Seaweeds are potential sources of several bioactive compounds with multiple applications in human health and environmental protection. Their composition includes substances such as vitamins, minerals, proteins, amino acids, sugars, lipids, terpenoids, polyphenols, polysaccharides, pigments, and enzymes with antibacterial, antioxidant, anti‐inflammatory, anticarcinogenic, and other pharmacological activities [8]. Due to their high biotechnological potential, macroalgae‐based extracts offer a fast, cost‐effective approach for discovering new compounds with antimicrobial properties that could help overcome the current antimicrobial resistance crisis [9]. Macroalgae‐derived metabolites have already been confirmed to exhibit bacteriostatic and bactericidal activities against gram‐positive and gram‐negative pathogens, mediated by multiple mechanisms of action, mostly involving alterations in cell wall and membrane structures and inhibition of metabolic enzymes. Therefore, effective extraction techniques, coupled with chemical characterization efforts, should be prioritized to increase the likelihood of uncovering chemical novelty in macroalgae specimens in these prospecting research efforts [9, 10].

*Gracilaria caudata* is a marine red macroalga (phylum Rhodophyta) distributed geographically in tropical and temperate regions [11] and is of major economic interest along the Brazilian coast [12]. It is well known as a source of agar, which is collected and sold by fishermen. Although these seaweeds are typically processed through depigmentation and drying before use, recent studies have investigated their postprocessing chemical composition and nutritional value in order to assess their potential for food, pharmaceutical, and biotechnological applications [13], with the few reports demonstrating the presence of a polysaccharidic fraction enriched with alternating residues 3‐linked‐*β*‐d‐galactopyranose and 4‐linked‐3,6‐*α*‐l‐anhydrogalactose [14] and high sulfate content [15], whereas lipids appear to be mostly composed of different fatty acids methyl esters when compared with other *Gracilaria* species [16].

In this context, although the chemical makeup and biological activity can vary substantially, we evaluated the in vitro antimicrobial potential of crude methanolic extract of *Gracilaria caudata* (MeOHGc), characterizing the chemical profiles, extraction method applied, and biological properties against human pathogenic bacteria, contributing to the search for marine‐derived solutions, reinforcing the potential of marine macroalgae as sustainable sources of bioactive compounds.

## 2. Materials and Methods

### 2.1. Collection and Freeze‐Drying Macroalgae Specimens

Specimens of *G. caudata* were collected in March 2024 on the Brazilian Atlantic coast (Enseada dos Corais Reef, Cabo de Santo Agostinho, PE—08° 18 ^′^ 51.42 ^″^ S; 34° 56 ^′^ 49.02 ^″^ W, and Boa Viagem, Recife, PE—08° 06 ^′^ 14 ^″^ S, 34° 52 ^′^ 50 ^″^ W). All individuals were collected in reef pools at the end of the dry season, during low and spring tides. Species identification was confirmed at Museu Oceanográfico da Universidade Federal de Pernambuco. Given the well‐established taxonomy of *G. caudata* along the Brazilian coast, together with the precise collection date and geographic coordinates provided above, these records ensure the traceability of the material used in this study. The specimen was cleaned with MilliQ water, sent to the Natural Products Laboratory under refrigeration (IEAPM, Arraial do Cabo, Rio de Janeiro, Brazil) and frozen at −20°C. Afterwards, the macroalgae material was freeze‐dried by overnight lyophilization for 3 days.

### 2.2. Preparation of *G. caudata* Extracts

A total of 7.5 g of the freeze‐dried material was Soxhlet‐extracted into 250 mL of methanol (Isofar). The extraction cycles were continued until the system was saturated, in approximately eight cycles of 48 min each. Finally, the extract was concentrated by rotaevaporation and stored in light‐protected vials at −20°C for future analyses. Chlorophyll was removed where necessary by treating the methanol extract with hexane (Sigma‐Aldrich) in a separating funnel. The extracts were concentrated by placing the 250 mL beaker in a water bath set at 55°C until all the solvent had evaporated. The dried extract was kept in the refrigerator at 4°C for its future use in phytochemical analysis.

### 2.3. Identification of Phytochemical Compounds in *G. caudata* Methanolic Extract by Qualitative Method

Preliminary qualitative phytochemical screening was carried out following standard protocols of the Shrestha et al. [17] for sterols, terpenoids, tannins, polyphenols, saponins, and glycosides. A color change was observed when the test reagent was added to the prepared sample for each phytochemical test. The results were recorded as present or absent based on the test outcome. All experiments were done in triplicate.

### 2.4. Tentative Identification of Chemical Constituents in *G. caudata* Methanolic Extract by Gas Chromatography–Mass Spectrometry (GC‐MS)

GC‐MS analysis of *G. caudata* methanol extract was performed using an Agilent 8860 GC System coupled to an Agilent 5977D MSD mass spectrometer, following the approach delineated by [18]. The separation step was performed on a DB‐5 ms capillary column (30 m × 0.25 mm i.d., 0.25 *μ*m film thickness; Agilent Technologies, Santa Clara, California, United States) using helium as the carrier gas. An aliquot of extract (1 *μ*L) was injected in splitless mode. The oven temperature was initially set to 100°C (held for 1 min), then increased to 260°C at 4°C/min, and held for 10 min. The injector and detector temperatures were set at 250°C and 230°C, respectively. Mass spectra were acquired in full scan mode over a range of 50–550 m/z, using electron ionization (EI) at 70 eV. GC‐MS analyses were performed in technical duplicates to ensure reproducibility of peak detection and compound identification. Compound identification was based on comparison of the obtained spectra with those in the NIST mass spectral library (Version 2.4), considering similarity indices equal to or greater than 90% (SI ≥90%).

### 2.5. Screening of Antimicrobial Activity in MeOHGc Extract Using the Disc Diffusion Method

The antimicrobial activity of *G. caudata* methanol extract was assessed using the agar disc diffusion method, as described in the Clinical and Laboratory Standards Institute (CLSI) document M100, supplement S29 [19]; CLSI interpretive breakpoints apply to standardized antibiotic susceptibility testing and were not used to classify the activity of the crude extract. The Gram‐positive bacteria *S. epidermidis* ATCC 12228 and the Gram‐negative *Escherichia coli* ATCC 25922 were utilized as indicator bacteria in this assay. All bacterial strains were cultured on Mueller–Hinton agar (Difco) at 37°C for 24 h before testing. For the disc diffusion assay, Mueller–Hinton agar plates were inoculated with exponentially grown bacterial suspensions adjusted in NaCl solution (0.85%) according to the McFarland scale (0.5). After, sterile Whatman No. 1 paper discs (6 mm) were loaded with 20 *μ*L of MeOHGc, redissolved in methanol to final concentrations ranging from 0.125 to 0.75 mg/mL, and air‐dried for 15 min at room temperature before being placed onto the inoculated agar surface. Considering the fixed loading volume of 20 *μ*L, the tested concentrations corresponded to 2.5, 5, 10, and 15 *μ*g of extract per disc (0.125, 0.25, 0.5, and 0.75 mg/mL, respectively). Solvent‐treated discs were prepared as a negative control, and vancomycin disc was used as positive control for Gram‐positive and ciprofloxacin disc for Gram‐negative. These discs were then applied onto the surface of the inoculated Mueller–Hinton agar. Later, plates were incubated at 37°C for 18–24 h, and the inhibitory zones were measured and expressed in millimeters (mm). Three replicates were conducted in the assay to ensure reproducibility.

### 2.6. Statistical Analysis

The results of zone inhibition (mm) in the antimicrobial assay were expressed as mean ± standard deviation of three replicates. The effect of the extract was checked by the one‐way ANOVA, followed by post hoc Tukey′s test for multiple comparisons between samples (*p* ≤ 0.05). All statistical tests were performed using the GraphPad Prism9 Software (GraphPad Software, Inc.).

## 3. Results and Discussion

### 3.1. Extraction, Qualitative Phytochemical Screening, and GC‐MS Analysis of the Extract

After all extraction cycles were completed, the methanolic extraction yielded 4.66% *w*/*w* relative to the dry weight of the algae material. Preliminary phytochemical screening of the MeOHGc demonstrated the presence of steroids, terpenoids, tannins, and phenolic compounds, as confirmed by positive reactions in the respective assays. Conversely, saponins and glycosides were not detected, as indicated by negative test results. Because Soxhlet extraction involves prolonged heating, some thermolabile compounds may have undergone partial degradation. Additionally, the hexane wash step, although effective for chlorophyll removal, may have inadvertently removed nonpolar bioactive lipids. Therefore, the chemical profile reported here likely reflects primarily the heat‐stable and polar constituents of the biomass. These results provide an initial overview of the major classes of secondary metabolites present in the extract. *Gracilaria* species are not only major sources of agar but also contain carbohydrates, proteins, lipids, minerals, and diverse bioactive compounds, including polyphenols, alkaloids, polyketides, cyclic peptides, phlorotannins, sterols, diterpenoids, quinones, and glycerols [20]. According to these phytochemical results, the presence of sterols, terpenoids, tannins, and polyphenols, and the absence of saponins and glycosides, may be associated with the abiotic conditions prevailing during the harvesting of the macroalga [21]. As discussed by Tan [21], in studies on brown macroalgae, the production and accumulation of secondary metabolites are strongly influenced by environmental factors such as light intensity, temperature, hydrodynamics, and nutrient availability, and these factors may also be relevant to other macroalgal species.

GC–MS analysis of the MeOHGc revealed between 15 and 25 distinct peaks, indicating a complex mixture of chemical constituents (Figure 1). Out of the 22 peaks detected in the chromatogram, two compounds were tentatively identified based on similarity index (SI ≥ 90%), corresponding to signals observed with the most intense responses occurring predominantly between 19 and 24 min of retention time (RT). The remaining peaks, although detected at lower intensities, likely represent additional constituents present in minor concentrations. The two compounds were benzenepropanoic acid, 3,5‐bis (1,1‐dimethylethyl)‐4‐hydroxy‐methyl ester (C_18_H_28_O_3_) in 19.03 min of RT (SI: 96.8%), and phenol, 2,4‐bis (1‐methyl‐1‐phenylethyl)‐ (SI: 96.8%) in 24.04 min of RT.

**Figure 1:**
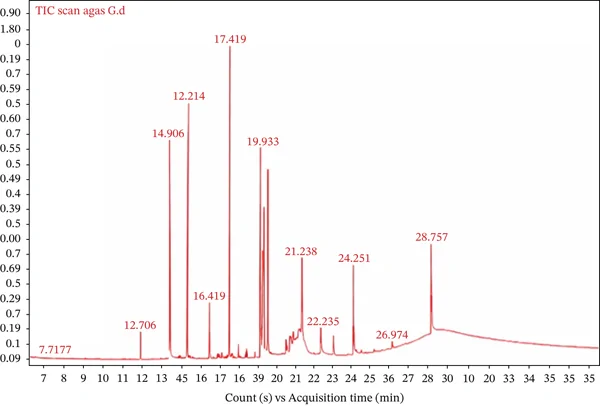
Gas chromatography–mass spectrometry (GC–MS) chromatogram of the methanolic extract of *Gracilaria caudata*, illustrating the chemical complexity of the extract and the chemical constituents tentatively identified based on similarity index values (SI ≥ 90%), benzenepropanoic acid, 3,5‐bis (1,1‐dimethylethyl)‐4‐hydroxy‐methyl ester (C_18_H_28_O_3_) in 19.03 min of RT (SI: 96.8%), and phenol, 2,4‐bis (1‐methyl‐1‐phenylethyl)‐ (SI: 96.8%) in 24.04 min of RT.

According to this, GC/MS analysis discloses the presence of ester benzene propanoic acid, 3,5‐bis (1,1‐dimethylethyl)‐4‐hydroxy‐ methyl ester (96.8% SI), which is already known for antifungal and antioxidant properties [22]. Nazarudin et al. [23] have reported its presence in the methanolic extract of *Sargassum polycystum,* a brown seaweed, where it was identified by GC‐MS with a comparable RT, reinforcing the consistency of our identification and supporting its occurrence in marine macroalgae.

Phenolic compounds, including benzoic acid, salicylic acid, gallic acid, p‐hydroxybenzoic acid, gentisic acid, syringic acid, protocatechuic acid, and vanillic acid, have been identified in the genus *Gracilaria* [24]. In our case, we detected phenol, 2,4‐bis (1‐methyl‐1‐phenylethyl), which has already been isolated from a fungal source by Choi and Ahsan [25] and has successfully suppressed bacterial growth of *Dickeya chrysanthemi* (syn. *Erwinia chrysanthemi*), a gram‐negative bacillus belonging to the family Pectobacteriaceae.

This chemical diversity confirms red algae as valuable multiproduct resources with broad applications. The *Gracilaria* spp. contain substances in the form of halogenated active compounds with haloforms, halogenated alkanes and alkenes, alcohols, aldehydes, hydroquinones, ketones, phenol, polyphenol, terpenes, and phlorotannins, which are also associated with antimicrobial activity [26]. However, recently untargeted metabolomic profiling of *G. caudata* under cadmium stress identified a distinct set of cellular metabolites dominated by primary metabolic intermediates, including monosaccharides, carboxylic acids, and amino acids that reflect physiological responses to environmental perturbation rather than the secondary metabolites typically implicated in antimicrobial effects [27]. This contrast underscores that different analytical approaches, such as targeted extract profiling versus comprehensive intracellular metabolomics, capture complementary facets of algal biochemistry. Although targeted profiling of organic extracts emphasizes lipophilic and semivolatile compounds with potential bioactivity, metabolomics reveals core metabolic adjustments to stress, indicating that secondary metabolite richness in *G. caudata* may not be directly reflected in whole‐cell metabolomes but remains accessible through extract‐based analyses. Together, these perspectives reinforce the importance of integrating multiple chemical profiling strategies to fully understand the bioactive potential of *G. caudata* and other red macroalgae.

### 3.2. Antibacterial Activity of *G. caudata* Methanolic Extract

The results of the antimicrobial activity analysis of the MeOHGc are shown in Figure 2. The MeOHGc showed antibacterial activity only against *S. epidermidis* at the tested concentrations. No inhibitory activity was produced by the solvent employed as a negative control. No measurable inhibition zone was observed at the lowest concentration tested (0.125 mg/mL). However, growth inhibition zones of *S. epidermidis* were observed at concentrations of 0.25, 0.5, and 0.75 mg/mL of the MeOHGc extract. As shown in Figure 2, the inhibition zone was significantly larger (*p* < 0.05) at 0.75 mg/mL (11.2 ± 0.4 mm). In contrast, there was no significant difference between the two lower concentrations, both showing slightly inhibited zones with diameters of 9.1 ± 0.26 mm (0.5 mg/mL) and 8.13 ± 0.66 mm (0.25 mg/mL). No antimicrobial activity was observed against *E. coli* at any tested concentration.

**Figure 2:**
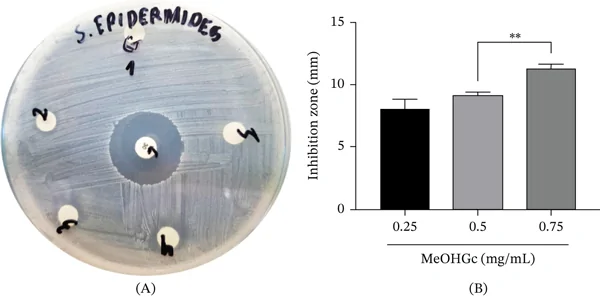
(A) Disc diffusion test of crude methanolic *G. caudata* against *Staphylococcus epidermidis*, disc 1 = 0.125 mg/mL; disc 2 = 0.25 mg/mL; disc 3 = 0.5 mg/mL; disc 4 = 0.75 mg/mL; disc 5 = solvent control; vancomycin disc at center as positive control. (B) Effects of *G. caudata* methanolic extract concentrations on antibacterial activity against *S. epidermidis*. The diameter of the inhibition zone was expressed in millimeters (mm); ∗∗*p* < 0.05.

Previous studies using *Gracilaria* extracts have reported similar results against Gram‐positive and Gram‐negative bacteria. Among the extracts obtained with solvents such as acetone, chloroform, and ethanol, the *G. corticata* methanolic extract showed the largest inhibition zone against Gram‐positive and Gram‐negative bacteria [28]. Likewise, the methanolic *G. edulis* extract was slightly more effective than the ethanol extract against *E. coli* and *S. aureus*, suggesting that methanol can extract bioactive compounds with antibacterial activity from marine algae [29]. However, some reports show the opposite. For example, a study investigated the antimicrobial potential of three solvents for *G. edulis* and *G. corticata* extracts, and the result demonstrated that ethyl acetate and butanol extracts produced significantly higher zone inhibition than methanolic extracts against *P. aeruginosa*, *S. aureus*, and *E. coli*, with the *G. corticata* methanolic extract being ineffective against all pathogens [30]. Another study using *G. opuntia* reported that the aqueous extract showed superior antimicrobial activity compared with methanol and ethanol extracts against pathogenic Gram‐positive and Gram‐negative bacteria [31].

These different results could rely on the most suitable solvent for the most effective extraction of algae, in order to fully exploit their antioxidant and antibacterial properties, which have not yet been defined. As demonstrated, Kurniawati et al. [32] reported that all red seaweeds, including *G. verrucosa*, exhibited antibacterial activity against *Enterobacter aerogenes*, *Salmonella enterica* serovar. Paratyphi, and *S. epidermidis*, findings similar to our results with *G. caudata*.

Therefore, the level of inhibition is dependent on the eluent of extraction and the microbial properties are affected by several factors, such as the habitats, seaweed cultivation methods, the age of extracted seaweed, the method used for the extraction of the bioactive components and even the season when seaweed is harvested, which can explain the lack of activity against *E. coli*.

## 4. Conclusion

Our findings suggest that MeOHGc have preliminary antibacterial potential, particularly against *S. epidermidis.* The GC‐MS analysis revealed the tentative presence of two chemical constituents, including benzenepropanoic acid, 3,5‐bis (1,1‐dimethylethyl)‐4‐hydroxy‐methyl ester and phenol, 2,4‐bis (1‐methyl‐1‐phenylethyl), compounds previously reported in other macroalgal species. As can be seen, species of this genus have been extensively studied because of the high utilization of their phycocolloids, but there are more biotechnological applications from *G. caudata*, and our previous results reveal that. Given the constant demand for new antibacterial agents, these preliminary findings support further investigation of *G. caudata* extracts as a candidate source of antibacterial compounds, pending compound isolation and confirmatory testing. Finally, more studies, including MIC and MBC determination, need to be conducted to assess the antimicrobial potential of the extract against other bacterial species and to determine its safety through in vitro and in vivo cytotoxicity, genotoxicity and hemolysis tests for future applications in cosmetics and other pharmaceuticals.

## Author Contributions

**Mariana de Sousa Santos Hempel:** conceptualization, methodology, validation, investigation, writing – original draft. **Michael dos Anjos dos Santos:** conceptualization, methodology, validation, investigation, writing – original draft. **Richard Coelho de Araujo dos Santos:** conceptualization, methodology, validation, investigation, writing – original draft. **João Vitor dos Anjos Reis:** conceptualization, methodology, validation, investigation, writing – original draft. **Leonardo Grip Bom Amorim:** methodology. **Bruno Francesco Rodrigues de Oliveira:** writing – review and editing, supervision. **Lohengrin Dias de Almeida Fernandes:** writing – review and editing, supervision. **Sávio Henrique Calazans Campos:** writing – review and editing, supervision. **Leonardo Zambotti-Villela:** writing – review and editing, supervision. **Pio Colepicolo Neto:** writing – review and editing, supervision. **Ricardo Coutinho:** writing – review and editing, supervision, project administration.

## Funding

This study was supported by the Coordenação de Aperfeiçoamento de Pessoal de Nível Superior (10.13039/501100002322, 88887.841475/2023‐00, 88887.991530/2024‐00, 88887.991940/2024‐00).

## Conflicts of Interest

The authors declare no conflicts of interest.

## Data Availability

The data that support the findings of this study are available on request from the corresponding author. The data are not publicly available due to privacy or ethical restrictions.
